# Synthesis and crystal structures of 1-benzoyl-4-(4-nitro­phen­yl)piperazine and 1-(4-bromo­benzo­yl)-4-phenyl­piperazine at 90 K

**DOI:** 10.1107/S2056989022009008

**Published:** 2022-09-22

**Authors:** Sreeramapura D. Archana, Haruvegowda Kiran Kumar, Holehundi J. Shankara Prasad, Hemmige S. Yathirajan, Sean Parkin

**Affiliations:** a Department of Studies in Chemistry, University of Mysore, Manasagangotri, Mysore-570 006, India; b Department of Chemistry, Yuvaraja’s College, University of Mysore, Mysore-570 005, India; c Department of Chemistry, University of Kentucky, Lexington, KY, 40506-0055, USA; University of Neuchâtel, Switzerland

**Keywords:** piperazine, crystal structure, absolute structure, aggregate crystal

## Abstract

Synthesis and crystal structures of 1-benzoyl-4-(4-nitro­phen­yl)piperazine, C_17_H_17_N_3_O_3_, (**I**) and 1-(4-bromo­benzo­yl)-4-phenyl­piperazine, C_17_H_17_BrN_2_O, (**II**) are described. Compounds **I** and **II** crystallize in the ortho­rhom­bic and monoclinic crystal systems with space groups *Pna*2_1_ (*Z*′ = 2, **I**) and *P*2_1_ (*Z*′ = 1, **II**), respectively. The crystal of **II** was a two-component aggregate, treated as a ‘twin’ for data-acquisition purposes. There are no conventional hydrogen bonds in either **I** or **II**, but there are weaker C—H⋯O contacts. Each mol­ecule consists of a central piperazine ring in a chair conformation, with either benzoyl and nitro­phenyl (**I**) or 4-bromo­benzoyl and phenyl (**II**) groups attached to different nitro­gen atoms of the piperazine. The various atom–atom contact coverages as qu­anti­fied by Hirshfeld surface analysis fingerprint plots are given.

## Abstract 2

The syntheses and low-temperature (90 K) crystal structures of 1-benzoyl-4-(4-nitro­phen­yl)piperazine and 1-(4-bromo­benzo­yl)phenyl­piperazine are presented.

## 1. Chemical context

Piperazines are important pharmacophores that are found in many biologically active compounds across a number of different therapeutic areas (Berkheij *et al.*, 2005 ▸; Brockunier *et al.*, 2004 ▸; Bogatcheva *et al.*, 2006 ▸) such as anti­fungal (Upadhayaya *et al.*, 2004 ▸), anti-bacterial, anti-malarial and anti-psychotic agents (Chaudhary *et al.*, 2006 ▸). The pharmacological properties of phenyl­piperazines and their derivatives have been described by Cohen *et al.* (1982 ▸), Conrado *et al.* (2008 ▸), Neves *et al.* (2003 ▸), and by Hanano *et al.* (2000 ▸). The design and synthesis of phenyl­piperazine derivatives as potent anti­cancer agents for prostate cancer have been described by Demirci *et al.* (2019 ▸). Many pharmaceutical compounds are derived from 1-phenyl­piperazine, *viz.*, oxypertine, trazodone, nefazodone, *etc*. Valuable insights into recent advances in anti­microbial activity of piperazine derivatives have been provided by Kharb *et al.* (2012 ▸). A review of current pharmacological and toxicological information for piperazine derivatives was conducted by Elliott (2011 ▸).

4-Nitro­phenyl­piperazinium chloride monohydrate has been used as an inter­mediate in the synthesis of anti­cancer drugs, transcriptase inhibitors and anti­fungal reagents, and is also an important reagent for potassium channel openers, which show considerable biomolecular current-voltage rectification characteristics (Lu, 2007 ▸). The inclusion behaviours of 4-sulf­on­ato­calix[*n*]arenes (SCX*n*) (*n* = 4, 6, 8) with 1-(4-nitro­phen­yl)piperazine (NPP) were investigated by UV and fluorescence spectroscopies at different pH values (Zhang *et al.*, 2014 ▸). The design, synthesis and biological profiling of aryl piperazine-based scaffolds for the management of androgen-sensitive prostatic disorders has also been reported by Gupta *et al.* (2016 ▸). 4-Nitro­phenyl­piperazine was the starting material in the synthesis and biological evaluation of novel piperazine containing hydrazone derivatives (Kaya *et al.*, 2016 ▸).

In view of the importance of piperazines in general and the use of 4-nitro­phenyl­piperazine and 1-phenyl­piperazine in particular, this paper reports the synthesis and crystal structures of 1-benzoyl-4-(4-nitro­phen­yl)piperazine, C_17_H_17_N_3_O_3_, (**I**) and 1-(4-bromo­benzo­yl)phenyl­piperazine, C_17_H_17_BrN_2_O, (**II**). 
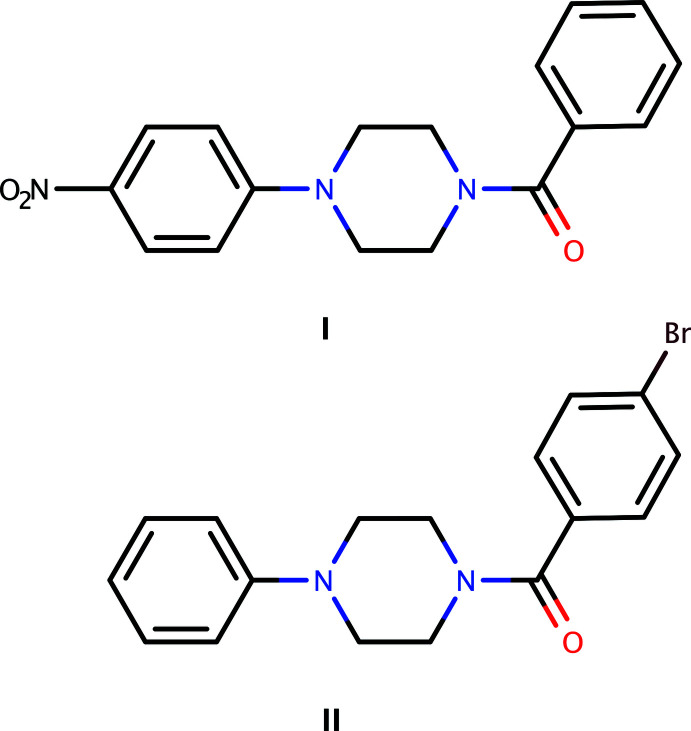


## 2. Structural commentary

**Figure 1:**
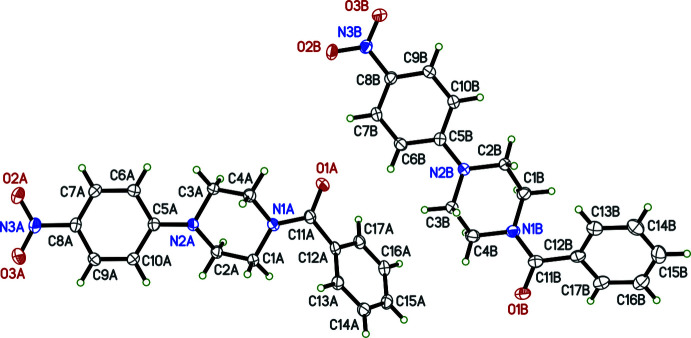
An ellipsoid plot (50% probability) of **I** showing the two mol­ecules in the asymmetric unit.

**Figure 2:**
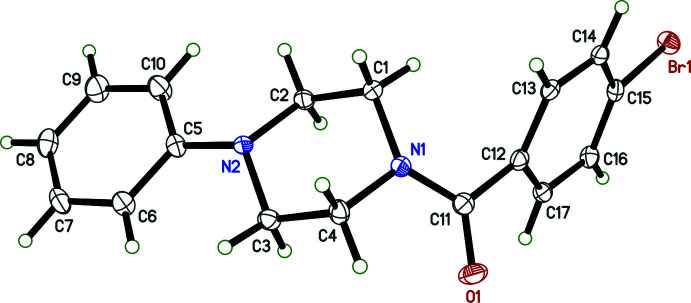
An ellipsoid plot (50% probability) of **II**.

There are no unusual bond distances or angles in either **I** or **II**. The asymmetric unit of **I** (see scheme) contains two mol­ecules, suffixed ‘*A*’ and ‘*B*’ in Fig. 1 ▸. Each consists of a central piperazine ring in a chair conformation, with a benzoyl and nitro­phenyl group attached to different nitro­gen atoms. The nitro groups are almost coplanar with their attached benzene rings, forming dihedral angles of 4.4 (2) and 3.0 (2)° for mol­ecules *A* and *B*, respectively. The phenyl rings are twisted out of planarity with the carbonyl group and its linkage to the piperazine rings, giving N1—C11—C12—C13 torsion angles of −46.8 (3) and 45.4 (3)° for *A* and *B*, respectively. The dihedral angles between the phenyl and nitro­benzene rings are 51.52 (6)° in *A* and 57.23 (7)° in *B*. Compound **II** on the other hand has just one mol­ecule in its asymmetric unit (Fig. 2 ▸). The piperazine ring is also in a chair conformation and the brominated ring is torsioned [N1—C11—C12—C13 = 46.4 (4)°] to a similar degree to that in **I**, but the dihedral angle between the phenyl and brominated benzene rings is larger, at 86.6 (1)°.

## 3. Supra­molecular features

**Figure 3:**
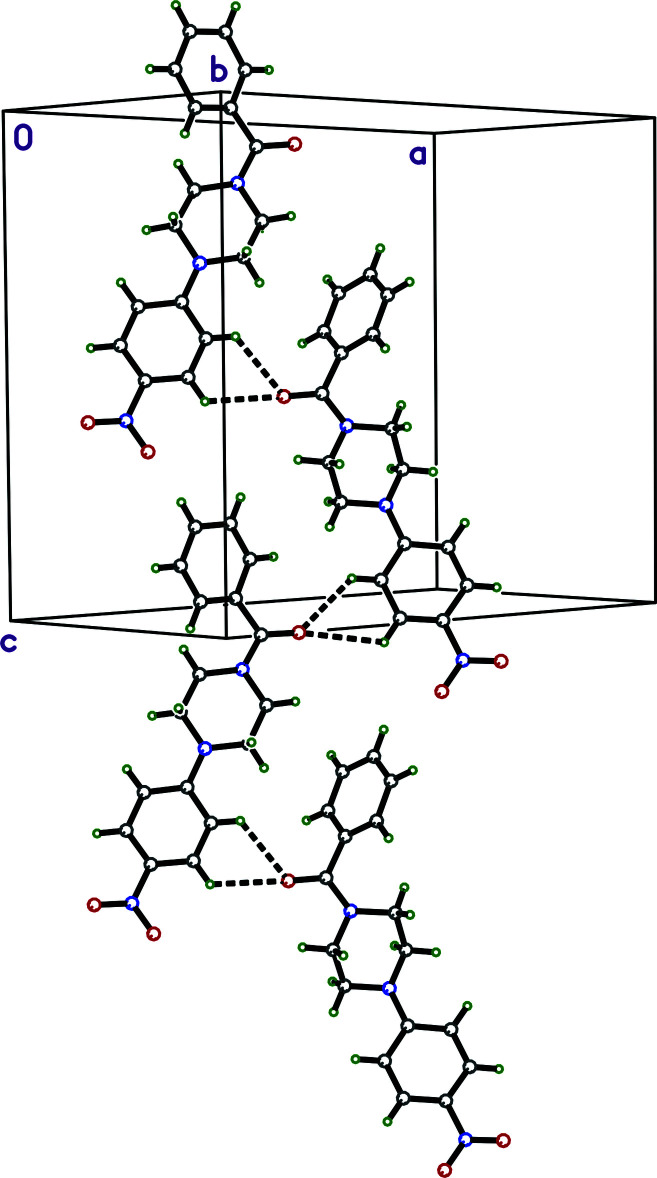
A partial packing plot of **I**, showing close contacts (dashed lines) that connect the mol­ecules into chains parallel to the *c*-axis.

**Figure 4:**
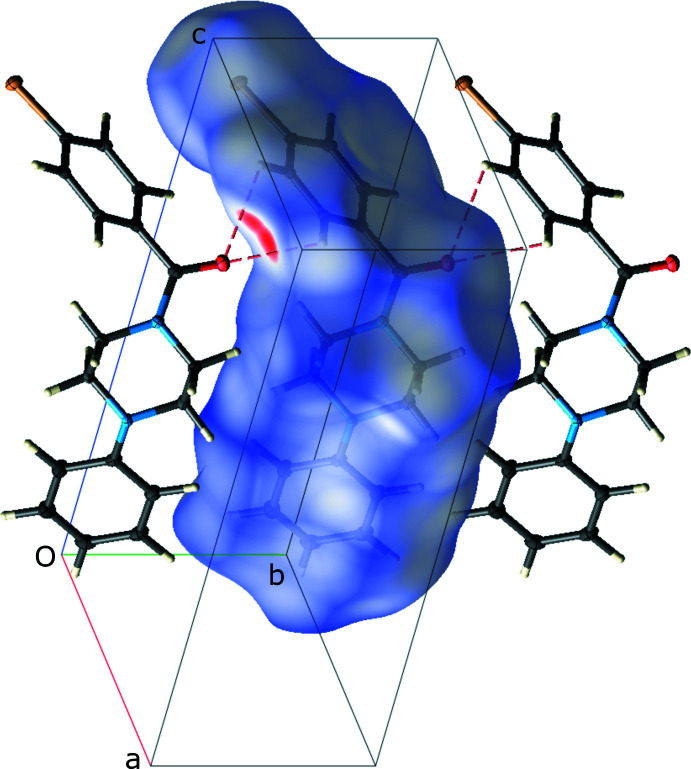
A partial packing plot of **II**, showing the Hirshfeld surface of the central mol­ecule, highlighting (red blobs) the bifurcated close contacts (dashed lines) that join the mol­ecules into chains parallel to the *b*-axis.

**Figure 5:**
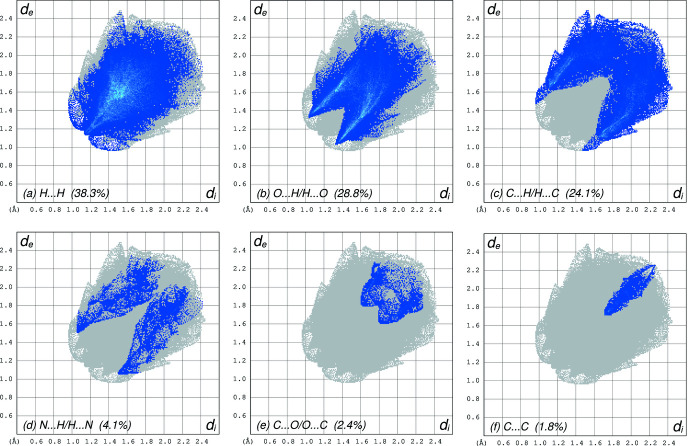
Hirshfeld surface analysis fingerprint plots showing the relative coverage of different atom-atom contacts in **I**: (*a*) H⋯H = 38.3%, (*b*) O⋯H/H⋯O = 28.8%, (*c*) C⋯H/H⋯C = 24.1%, (*d*) N⋯H/H⋯N = 4.1%, (*e*) C⋯O/O.·C = 2.4%, (*f*) C⋯C = 1.8%. All other contacts are negligible.

There are no conventional hydrogen bonds in either **I** or **II**, but there are weaker C—H⋯O contacts (Table 1 ▸). For **I**, *SHELXL* identifies a number of ‘potential’ hydrogen-bonding inter­actions, but most of these have poor geometry for hydrogen bonds. The shortest donor–acceptor distances occur for the bifurcated pair C6*B*—H6*B*⋯O1*A* and C7*B*—H7*B*⋯O1*A* within the chosen asymmetric unit. A similar bifurcated pair of contacts C6*A*—H6*A*⋯O1*B* ^i^ and C7*A*—H7*A*⋯O1*B* ^i^ [symmetry code: (i) *x*, *y*, *z* + 1] occur between the *A* and *B* mol­ecules in adjacent (along *c*) asymmetric units. In combination, these inter­actions lead to double chains that extend parallel to [001] (Fig. 3 ▸). In contrast to **I**, *SHELXL* identifies no ‘potential’ hydrogen bonds for **II**. *Mercury* (Macrae *et al.*, 2020 ▸) on the other hand, which has different default parameters for flagging hydrogen bonds, identifies a bifurcated pair, C13—H13⋯O1^ii^ and C14—H14⋯O1^ii^ [symmetry code: (ii) *x*, *y* + 1, *z*] (Table 1 ▸). A clearer picture of this inter­action is provided by a view of the Hirshfeld surface plotted over *d*_norm_, as calculated by *CrystalExplorer* (Spackman *et al.*, 2021 ▸), which highlights contacts shorter than the van der Waals radius sum as red blobs (Fig. 4 ▸). This bifurcated pair of inter­actions link mol­ecules of **II** into chains that extend along [010]. The various atom–atom contacts as qu­anti­fied in Hirshfeld surface analysis fingerprint plots are given in Figs. 5 ▸ and 6 ▸.

## 4. Database survey

There are numerous crystal structures related to **I** and **II** in the Cambridge Structure Database (CSD v5.42 with updates through June 2022; Groom *et al.*, 2016 ▸). A search on the central core, piperazine-1-carbaldehyde gave 834 hits whereas search fragments 4-benzoyl­piperazine and 4-phenyl­piperazine-1-carbaldehyde gave 132 and 110 hits, respectively. A search on 1-benzoyl-4-phenyl­piperazine gave 20 hits, two of which have little in common with **I** or **II**. An NMR-based investigation of conformational behaviour in solution by Wodtke *et al.* (2018 ▸) of acyl-functionalized piperazines includes the crystal structures of 1-(4-fluoro­benzo­yl)-4-(4-nitro­phen­yl)piperazine (BIQYIM), 1-(4-bromo­benzo­yl)-4-(4-nitro­phen­yl)piperazine (BIRHES), and 1-(3-bromo­benzo­yl)-4-(4-nitro­phen­yl)piperazine (BIRHIW). Six new 1-aroyl-4-(4-meth­oxy­phen­yl)piperazines (VONFOW, VONGAJ, VONGEN, VONGIR, VONGOX, VONGUD) were prepared using coupling reactions between benzoic acids and *N*-(4-meth­oxy­phen­yl)piperazine (Kiran Kumar *et al.*, 2019 ▸). Six 1-halobenzoyl-4-(2-meth­oxy­phen­yl)piperazines (FALHEJ, FALHIN, FALHOT, FALHUZ, FALJAH, FALJEL) with a variety of disorder, pseudosymmetry and twinning were described by Harish Chinthal *et al.* (2021 ▸). 1-(3,5-Di­nitro­benzo­yl)-4-(2-meth­oxy­phen­yl)piperazine (LAHBIJ) was published by Harish Chinthal *et al.* (2020 ▸). The remaining two hits are piperazine derivatives with (2-meth­oxy­phenyl­sulfan­yl)benzoyl groups plus 2,3-di­chloro­phenyl (DEGHAZ: Chu *et al.*, 2006 ▸) and 2-meth­oxy­phenyl (SAYYEX: Li *et al.*, 2006 ▸).

## 5. Synthesis and crystallization

**Figure 7:**
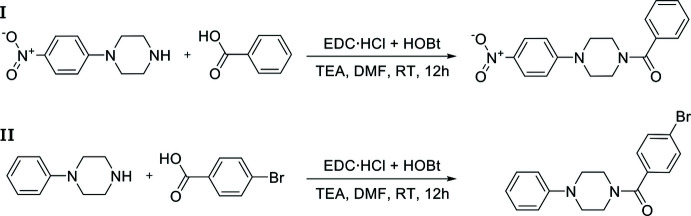
Reaction schemes for the synthesis of **I** and **II**. EDC·HCl = 1-(3-di­methyl­amino­prop­yl)-3-ethyl­carbodi­imide hydro­chloride, HOBt = 1-hy­droxy­benzotriazole, TEA = tri­ethyl­amine, DMF = di­methyl­formamide.

Synthetic routes for compounds similar to **I** and **II** have already been reported by two separate research groups (Kumari *et al.*, 2015 ▸; Wodtke *et al.*, 2018 ▸). The present syntheses are totally different from those earlier reports. 1-(3-Di­methyl­amino­prop­yl)-3-ethyl­carbodi­imide hydro­chloride (109 mg, 0.7 mmol), 1-hy­droxy­benzotriazole (68 mg, 0.5 mmol) and tri­ethyl­amine (0.5 ml, 1.5 mmol) were added to a solution of benzoic acid (0.5 mmol) or 4-bromo­benzoic acid (0.5 mmol) in *N*,*N*-di­methyl­formamide (5 ml) and the resulting mixture was stirred for 20 min at 273 K. A solution of 1-(4-nitro­phen­yl)piperazine (104 mg, 0.5 mmol) or 1-phenyl­piperazine (81 mg, 0.5 mmol) in *N*,*N*-di­methyl­formamide (5 ml) was then added and stirring was continued overnight at ambient temperature. Reaction schemes are summarized in Fig. 7 ▸. When the reactions were confirmed to be complete using thin-layer chromatography, each mixture was quenched with water (10 ml) and extracted with ethyl acetate (20 ml). Each organic fraction was separated and washed successively with an aqueous hydro­chloric acid solution (1 mol dm^−3^), a saturated solution of sodium hydrogencarbonate, and lastly with brine. The organic phases were dried over anhydrous sodium sulfate and the solvent was removed under reduced pressure. Crystals suitable for single-crystal X-ray diffraction were grown by slow evaporation, at ambient temperature and in the presence of air, of solutions in ethyl acetate (**I**: yield 81%, m.p. 428–430 K; **II**: yield 75%, m.p. 394–396 K).

## 6. Data collection and structure refinement

For **I**, an orange, irregular block-shaped crystal was mounted using polyisobutene oil on the tip of a fine glass fibre in a copper mounting pin. Cu *Kα* radiation was chosen to facilitate setting the correct absolute structure, which was definitively established by variants of Flack’s parameter (Flack & Bernardinelli, 1999 ▸; Hooft *et al.*, 2008 ▸; Parsons *et al.*, 2013 ▸). For **II**, the available sample consisted of colourless plates, none of which were single crystals. A suitable specimen was mounted in the same way as for **I**. Diffraction data collected at 90 K showed two slightly mis-aligned, but sharp and distinct reciprocal lattices. These were not related by any rational twin operation, but by a seemingly arbitrary ∼4° rotation, presumably due to mis-stacking of aggregated plates. Nevertheless, for data acquisition and processing, facilities for handling twinning by non-merohedry were used. For a brief discussion of true twins *vs* aggregates, see Parkin (2021 ▸). The absolute structure was again determined unambiguously *via* the Flack parameter and related methods. Crystal data, data collection and refinement statistics are summarized in Table 2 ▸. For both structures, hydrogen atoms were included using riding models, with constrained distances set to 0.95 Å (C*sp* ^2^H) and 0.99 Å (*R*_2_CH_2_). *U*_iso_(H) parameters were set to 1.2*U*_eq_ of the attached atom.

## Supplementary Material

Crystal structure: contains datablock(s) I, II, global. DOI: 10.1107/S2056989022009008/tx2058sup1.cif

Structure factors: contains datablock(s) I. DOI: 10.1107/S2056989022009008/tx2058Isup2.hkl

Structure factors: contains datablock(s) II. DOI: 10.1107/S2056989022009008/tx2058IIsup3.hkl

CCDC references: 2205954, 2205953

Additional supporting information:  crystallographic information; 3D view; checkCIF report

## Figures and Tables

**Figure 6:**
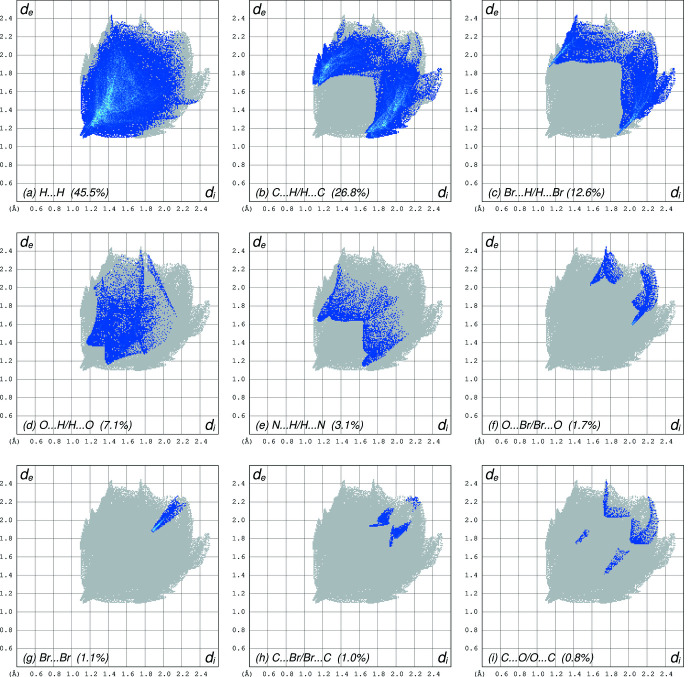
Hirshfeld surface analysis fingerprint plots showing the relative coverage of different atom-atom contacts in **II**: (*a*) H⋯H = 45.5%, (*b*) C⋯H/H⋯C = 26.8, (*c*) Br⋯H/H⋯Br = 12.6%, (*d*) O⋯H/H⋯O = 7.1%, (*e*) N⋯H/H.·N = 3.1%, (*f*) O⋯Br/Br⋯O = 1.7%, (*g*) Br⋯Br = 1.1%, (*h*) C⋯Br/Br⋯C = 1.0%, (i) C⋯O/O⋯C = 0.8%. All other contacts are negligible.

**Table 1:** Short inter­molecular C—H⋯O contacts (Å, °) in **I** and **II**

*D*—H⋯*A*	*D*—H	H⋯*A*	*D*⋯*A*	*D*—H⋯*A*
**I**				
C6*B*—H6*B*⋯O1*A*	0.95	2.50	3.140 (2)	124.5
C7*B*—H7*B*⋯O1*A*	0.95	2.58	3.171 (2)	120.3
C6*A*—H6*A*⋯O1*B* ^i^	0.95	2.47	3.173 (2)	131.0
C7*A*—H7*A*⋯O1*B* ^i^	0.95	2.78	3.317 (2)	116.8
				
**II**				
C13—H13⋯O1^ii^	0.95	2.60	3.018 (4)	107.3
C14—H14⋯O1^ii^	0.95	2.68	3.052 (4)	104.0

Symmetry codes: (i) *x*, *y*, *z* + 1; (ii) *x*, *y* − 1, *z*

**Table 2:** Experimental details

	**I**	II
Crystal data		
Chemical formula	C_17_H_17_N_3_O_3_	C_17_H_17_BrN_2_O
*M*_r_	311.33	345.23
Crystal system, space group	Orthorhombic, *P* *n* *a*2_1_	Monoclinic, *P*2_1_
Temperature (K)	90	90
*a*, *b*, *c* (Å)	18.7779 (4), 10.0699 (2), 15.7288 (3)	7.5162 (3), 6.1125 (2), 15.7249 (5)
α, β, γ (°)	90, 90, 90	90, 98.625 (1), 90
*V* (Å^3^)	2974.18 (10)	714.28 (4)
*Z*	8	2
Radiation type	Cu *K*α	Mo *K*α
μ (mm^−1^)	0.80	2.88
Crystal size (mm)	0.24 × 0.18 × 0.12	0.35 × 0.20 × 0.06

Data collection		
Diffractometer	Bruker D8 Venture dual source	Bruker D8 Venture dual source
Absorption correction	Multi-scan (*SADABS*; Krause et al., 2015 ▸)	Multi-scan (*TWINABS*; Sheldrick, 2012 ▸)
*T*_min_, *T*_max_	0.854, 0.942	0.568, 0.806
No. of measured, independent and observed [*I* > 2σ(*I*)] reflections	24139, 5684, 5575	6918, 6918, 6410
*R*_int_	0.027	0.065
(sin θ/λ)_max_ (Å^−1^)	0.625	0.650

Refinement		
*R*[*F* ^2^ > 2σ(*F* ^2^)], *wR*(*F* ^2^), *S*	0.028, 0.075, 1.04	0.023, 0.049, 1.04
No. of reflections	5684	6918
No. of parameters	416	191
No. of restraints	1	1
H-atom treatment	H-atom parameters constrained	H-atom parameters constrained
Δρ_max_, Δρ_min_ (e Å^−3^)	0.18, −0.16	0.29, −0.22
Absolute structure	Flack *x* determined using 2442 quotients [(*I* ^+^)−(*I* ^−^)]/[(*I* ^+^)+(*I* ^−^)] (Parsons *et al.*, 2013 ▸)	Flack *x* determined using 1306 quotients [(*I* ^+^)−(*I* ^−^)]/[(*I* ^+^)+(*I* ^−^)] (Parsons *et al.*, 2013 ▸)
Absolute structure parameter	0.01 (5)	0.012 (4)

Computer programs: *APEX3* (Bruker, 2016 ▸), *SHELXT* (Sheldrick, 2015*a* ▸), *SHELXL2019/2* (Sheldrick, 2015*b* ▸), *SHELXTL* and *XP* in *SHELXTL* (Sheldrick, 2008 ▸) and *publCIF* (Westrip, 2010 ▸).

## supplementary crystallographic information

### 1-Benzoyl-4-(4-nitrophenyl)piperazine (I) Crystal data

**Table:**

C_17_H_17_N_3_O_3_	*D*_x_ = 1.391 Mg m^−^^3^
*M**_r_* = 311.33	Cu *K*α radiation, λ = 1.54178 Å
Orthorhombic, *P**n**a*2_1_	Cell parameters from 9920 reflections
*a* = 18.7779 (4) Å	θ = 4.4–74.3°
*b* = 10.0699 (2) Å	µ = 0.80 mm^−^^1^
*c* = 15.7288 (3) Å	*T* = 90 K
*V* = 2974.18 (10) Å^3^	Cut block, orange
*Z* = 8	0.24 × 0.18 × 0.12 mm
*F*(000) = 1312	

### 1-Benzoyl-4-(4-nitrophenyl)piperazine (I) Data collection

**Table:**

Bruker D8 Venture dual source diffractometer	5684 independent reflections
Radiation source: microsource	5575 reflections with *I* > 2σ(*I*)
Detector resolution: 7.41 pixels mm^-1^	*R*_int_ = 0.027
φ and ω scans	θ_max_ = 74.5°, θ_min_ = 4.7°
Absorption correction: multi-scan (*SADABS*; Krause et al., 2015)	*h* = −23→23
*T*_min_ = 0.854, *T*_max_ = 0.942	*k* = −12→11
24139 measured reflections	*l* = −17→19

### 1-Benzoyl-4-(4-nitrophenyl)piperazine (I) Refinement

**Table:**

Refinement on *F*^2^	Hydrogen site location: difference Fourier map
Least-squares matrix: full	H-atom parameters constrained
*R*[*F*^2^ > 2σ(*F*^2^)] = 0.028	*w* = 1/[σ^2^(*F*_o_^2^) + (0.0371*P*)^2^ + 0.6857*P*] where *P* = (*F*_o_^2^ + 2*F*_c_^2^)/3
*wR*(*F*^2^) = 0.075	(Δ/σ)_max_ < 0.001
*S* = 1.04	Δρ_max_ = 0.18 e Å^−^^3^
5684 reflections	Δρ_min_ = −0.15 e Å^−^^3^
416 parameters	Extinction correction: SHELXL2019/2 (Sheldrick 2015b), Fc^*^=kFc[1+0.001xFc^2^λ^3^/sin(2θ)]^-1/4^
1 restraint	Extinction coefficient: 0.0022 (4)
Primary atom site location: structure-invariant direct methods	Absolute structure: Flack *x* determined using 2442 quotients [(*I*^+^)-(*I*^-^)]/[(*I*^+^)+(*I*^-^)] (Parsons *et al.*, 2013)
Secondary atom site location: difference Fourier map	Absolute structure parameter: 0.01 (5)

### 1-Benzoyl-4-(4-nitrophenyl)piperazine (I) Special details

**Table:**

Geometry. All esds (except the esd in the dihedral angle between two l.s. planes) are estimated using the full covariance matrix. The cell esds are taken into account individually in the estimation of esds in distances, angles and torsion angles; correlations between esds in cell parameters are only used when they are defined by crystal symmetry. An approximate (isotropic) treatment of cell esds is used for estimating esds involving l.s. planes.
Refinement. Refinement progress was checked using *Platon* (Spek, 2020) and by an *R*-tensor (Parkin, 2000). The final model was further checked with the IUCr utility *checkCIF*.

### 1-Benzoyl-4-(4-nitrophenyl)piperazine (I) Fractional atomic coordinates and isotropic or equivalent isotropic displacement parameters (Å^2^)

**Table:**

	*x*	*y*	*z*	*U*_iso_*/*U*_eq_	
O1A	0.49268 (7)	0.25685 (14)	0.56553 (9)	0.0244 (3)	
O2A	0.68624 (9)	0.60163 (17)	1.17477 (10)	0.0361 (4)	
O3A	0.76060 (9)	0.73323 (17)	1.11319 (11)	0.0414 (4)	
N1A	0.55858 (8)	0.41895 (16)	0.62597 (10)	0.0210 (3)	
N2A	0.61000 (8)	0.49591 (17)	0.78914 (10)	0.0195 (3)	
N3A	0.71215 (9)	0.65057 (17)	1.11034 (11)	0.0261 (4)	
C1A	0.62179 (10)	0.5034 (2)	0.63281 (12)	0.0214 (4)	
H1AA	0.607617	0.597868	0.628176	0.026*	
H1AB	0.654911	0.483266	0.585577	0.026*	
C2A	0.65919 (10)	0.48038 (19)	0.71744 (12)	0.0207 (4)	
H2AA	0.679576	0.389723	0.718316	0.025*	
H2AB	0.698868	0.544445	0.723448	0.025*	
C3A	0.55049 (10)	0.4032 (2)	0.78203 (12)	0.0210 (4)	
H3AA	0.517626	0.415436	0.830622	0.025*	
H3AB	0.568435	0.310825	0.783299	0.025*	
C4A	0.51124 (10)	0.4282 (2)	0.69932 (12)	0.0226 (4)	
H4AA	0.472393	0.362465	0.693253	0.027*	
H4AB	0.489499	0.517774	0.700923	0.027*	
C5A	0.6365 (1)	0.52962 (18)	0.86888 (12)	0.0185 (4)	
C6A	0.61095 (10)	0.4696 (2)	0.94373 (13)	0.0212 (4)	
H6A	0.576303	0.401156	0.939965	0.025*	
C7A	0.63554 (10)	0.5089 (2)	1.02259 (13)	0.0224 (4)	
H7A	0.618067	0.467679	1.072742	0.027*	
C8A	0.68595 (10)	0.6092 (2)	1.02784 (13)	0.0210 (4)	
C9A	0.71253 (10)	0.67057 (19)	0.95518 (13)	0.0204 (4)	
H9A	0.746997	0.739266	0.959720	0.025*	
C10A	0.68827 (10)	0.63056 (19)	0.87663 (12)	0.0196 (4)	
H10A	0.706667	0.671542	0.826856	0.024*	
C11A	0.54106 (10)	0.33847 (18)	0.55996 (12)	0.0193 (4)	
C12A	0.58053 (10)	0.35251 (18)	0.47737 (12)	0.0192 (4)	
C13A	0.59294 (11)	0.47549 (19)	0.43979 (13)	0.0217 (4)	
H13A	0.580037	0.554491	0.468986	0.026*	
C14A	0.62417 (11)	0.4834 (2)	0.35964 (13)	0.0248 (4)	
H14A	0.632511	0.567619	0.334269	0.030*	
C15A	0.64312 (11)	0.3679 (2)	0.31679 (13)	0.0245 (4)	
H15A	0.664928	0.373155	0.262397	0.029*	
C16A	0.6302 (1)	0.24515 (19)	0.35343 (12)	0.0235 (4)	
H16A	0.643110	0.166306	0.324057	0.028*	
C17A	0.59843 (10)	0.23706 (19)	0.43299 (12)	0.0216 (4)	
H17A	0.588821	0.152610	0.457348	0.026*	
O1B	0.51435 (8)	0.27356 (15)	0.05162 (9)	0.0293 (3)	
O2B	0.33405 (8)	−0.06911 (15)	0.67739 (9)	0.0282 (3)	
O3B	0.26109 (8)	−0.20433 (15)	0.61629 (10)	0.0329 (4)	
N1B	0.45677 (9)	0.11868 (16)	0.12905 (11)	0.0228 (4)	
N2B	0.40172 (9)	0.04918 (17)	0.29159 (10)	0.0217 (3)	
N3B	0.30667 (8)	−0.11612 (16)	0.61295 (11)	0.0224 (3)	
C1B	0.41561 (12)	−0.0033 (2)	0.14059 (13)	0.0253 (4)	
H1BA	0.448370	−0.078227	0.151953	0.030*	
H1BB	0.388941	−0.023286	0.087827	0.030*	
C2B	0.36400 (11)	0.0112 (2)	0.21386 (13)	0.0234 (4)	
H2BA	0.328046	0.079722	0.199825	0.028*	
H2BB	0.338820	−0.073922	0.223114	0.028*	
C3B	0.44607 (11)	0.1676 (2)	0.28017 (13)	0.0233 (4)	
H3BA	0.473969	0.183527	0.332597	0.028*	
H3BB	0.415063	0.245682	0.270653	0.028*	
C4B	0.49644 (10)	0.1524 (2)	0.20567 (13)	0.0250 (4)	
H4BA	0.522715	0.236492	0.196672	0.030*	
H4BB	0.531553	0.081701	0.218093	0.030*	
C5B	0.37409 (10)	0.01813 (18)	0.37065 (13)	0.0188 (4)	
C6B	0.40552 (10)	0.06711 (19)	0.44607 (13)	0.0217 (4)	
H6B	0.442914	0.130351	0.442141	0.026*	
C7B	0.38332 (10)	0.02550 (19)	0.52486 (13)	0.0199 (4)	
H7B	0.405633	0.058671	0.574731	0.024*	
C8B	0.32797 (10)	−0.0655 (2)	0.53103 (13)	0.0215 (4)	
C9B	0.29260 (11)	−0.1085 (2)	0.45864 (14)	0.0291 (5)	
H9B	0.252897	−0.166422	0.463589	0.035*	
C10B	0.31504 (11)	−0.0671 (2)	0.37969 (13)	0.0286 (5)	
H10B	0.290331	−0.096502	0.330455	0.034*	
C11B	0.46938 (10)	0.1852 (2)	0.05581 (13)	0.0220 (4)	
C12B	0.42532 (10)	0.15513 (18)	−0.02141 (13)	0.0221 (4)	
C13B	0.35132 (11)	0.14223 (19)	−0.01901 (14)	0.0256 (4)	
H13B	0.327087	0.142772	0.034009	0.031*	
C14B	0.31314 (12)	0.1286 (2)	−0.09407 (15)	0.0306 (5)	
H14B	0.262847	0.118875	−0.091983	0.037*	
C15B	0.34732 (13)	0.1291 (2)	−0.17155 (15)	0.0322 (5)	
H15B	0.320685	0.120065	−0.222572	0.039*	
C16B	0.42102 (13)	0.1427 (2)	−0.17482 (14)	0.0299 (5)	
H16B	0.444836	0.143404	−0.228106	0.036*	
C17B	0.45955 (11)	0.15539 (19)	−0.10018 (14)	0.0256 (4)	
H17B	0.509876	0.164349	−0.102622	0.031*	

### 1-Benzoyl-4-(4-nitrophenyl)piperazine (I) Atomic displacement parameters (Å^2^)

**Table:**

	*U*^11^	*U*^22^	*U*^33^	*U*^12^	*U*^13^	*U*^23^
O1A	0.0266 (7)	0.0212 (7)	0.0255 (7)	−0.0069 (5)	−0.0025 (6)	−0.0020 (6)
O2A	0.0437 (9)	0.0473 (10)	0.0173 (7)	−0.0087 (7)	−0.0004 (7)	−0.0012 (7)
O3A	0.0493 (9)	0.0485 (10)	0.0263 (8)	−0.0226 (8)	−0.0077 (8)	−0.0045 (8)
N1A	0.0202 (8)	0.0234 (8)	0.0195 (8)	−0.0048 (6)	0.0003 (6)	−0.0035 (7)
N2A	0.0180 (7)	0.0237 (9)	0.0169 (8)	−0.0036 (6)	0.0013 (6)	−0.0035 (6)
N3A	0.0289 (8)	0.0304 (9)	0.0189 (8)	−0.0013 (7)	−0.0027 (7)	−0.0021 (7)
C1A	0.0228 (9)	0.0241 (10)	0.0173 (10)	−0.0060 (7)	0.0006 (7)	−0.0028 (7)
C2A	0.0190 (9)	0.0244 (9)	0.0188 (10)	−0.0021 (7)	0.0013 (7)	−0.0036 (8)
C3A	0.0188 (8)	0.0249 (10)	0.0194 (9)	−0.0062 (7)	0.0018 (7)	−0.0022 (8)
C4A	0.0193 (9)	0.0273 (10)	0.0213 (10)	−0.0036 (7)	0.0013 (8)	−0.0043 (8)
C5A	0.0170 (8)	0.0199 (9)	0.0185 (9)	0.0025 (7)	0.0000 (7)	−0.0021 (7)
C6A	0.0198 (9)	0.0218 (9)	0.0219 (10)	−0.0022 (7)	0.0009 (8)	−0.0001 (8)
C7A	0.0235 (9)	0.0255 (10)	0.0183 (9)	0.0000 (8)	0.0033 (8)	0.0008 (8)
C8A	0.0212 (9)	0.0243 (10)	0.0175 (9)	0.0027 (7)	−0.0005 (7)	−0.0035 (8)
C9A	0.0181 (8)	0.0200 (9)	0.0232 (10)	−0.0010 (7)	−0.0013 (8)	−0.0019 (8)
C10A	0.0194 (9)	0.0213 (9)	0.0182 (9)	0.0004 (7)	0.0018 (7)	0.0008 (7)
C11A	0.0215 (9)	0.0170 (9)	0.0193 (9)	0.0016 (7)	−0.0053 (7)	0.0004 (7)
C12A	0.0206 (9)	0.0187 (9)	0.0183 (9)	0.0000 (7)	−0.0064 (7)	−0.0027 (7)
C13A	0.0262 (9)	0.0198 (9)	0.0192 (9)	0.0008 (7)	−0.0034 (8)	−0.0007 (7)
C14A	0.027 (1)	0.0231 (10)	0.0243 (11)	−0.0019 (8)	−0.0061 (8)	0.0034 (8)
C15A	0.0224 (10)	0.0327 (11)	0.0185 (10)	0.0009 (8)	−0.0033 (7)	−0.0007 (8)
C16A	0.0233 (9)	0.0255 (10)	0.0218 (11)	0.0043 (8)	−0.0047 (8)	−0.0055 (8)
C17A	0.0244 (9)	0.0188 (9)	0.0215 (10)	0.0008 (7)	−0.0058 (7)	−0.0001 (7)
O1B	0.0278 (7)	0.0310 (8)	0.0290 (8)	−0.0081 (6)	0.0022 (6)	0.0076 (6)
O2B	0.0288 (7)	0.0390 (8)	0.0169 (7)	−0.0015 (6)	−0.0034 (6)	−0.0029 (6)
O3B	0.0366 (8)	0.0368 (8)	0.0255 (8)	−0.0143 (7)	0.0050 (7)	−0.0004 (7)
N1B	0.0261 (8)	0.0203 (8)	0.0221 (9)	−0.0031 (7)	0.0031 (6)	0.0008 (7)
N2B	0.0228 (8)	0.0239 (8)	0.0183 (8)	−0.0061 (7)	−0.0002 (6)	−0.0008 (6)
N3B	0.0220 (7)	0.0263 (8)	0.0189 (8)	0.0012 (6)	0.0009 (7)	−0.0011 (7)
C1B	0.0353 (11)	0.0183 (9)	0.0224 (10)	−0.0035 (8)	0.0039 (8)	−0.0009 (7)
C2B	0.0285 (10)	0.024 (1)	0.0176 (10)	−0.0089 (8)	0.0001 (8)	−0.0007 (8)
C3B	0.0253 (9)	0.0245 (10)	0.0203 (10)	−0.0070 (8)	0.0000 (8)	−0.0004 (8)
C4B	0.0220 (9)	0.0263 (10)	0.0266 (11)	−0.0026 (7)	−0.0001 (8)	0.0035 (8)
C5B	0.0186 (9)	0.0188 (9)	0.0189 (9)	0.0028 (7)	−0.0006 (7)	−0.0011 (7)
C6B	0.0200 (8)	0.0214 (9)	0.0236 (9)	−0.0025 (7)	−0.0005 (8)	−0.0024 (8)
C7B	0.0199 (9)	0.0204 (10)	0.0194 (9)	0.0003 (7)	−0.0035 (7)	−0.0041 (7)
C8B	0.0209 (9)	0.0248 (10)	0.0187 (9)	0.0004 (8)	0.0019 (7)	−0.0014 (8)
C9B	0.0268 (10)	0.0377 (12)	0.0227 (10)	−0.0135 (9)	0.0010 (9)	−0.0025 (9)
C10B	0.0263 (10)	0.0397 (12)	0.0197 (10)	−0.0116 (9)	−0.0011 (8)	−0.0035 (9)
C11B	0.0206 (9)	0.0196 (9)	0.0257 (10)	0.0019 (7)	0.0052 (8)	0.0007 (8)
C12B	0.0269 (10)	0.0136 (9)	0.0258 (10)	0.0013 (7)	0.0041 (8)	0.0028 (7)
C13B	0.0264 (10)	0.0194 (10)	0.0311 (12)	0.0011 (8)	0.0035 (8)	−0.0021 (8)
C14B	0.0300 (11)	0.022 (1)	0.0398 (12)	−0.0012 (8)	−0.0013 (10)	−0.0051 (9)
C15B	0.0452 (13)	0.0194 (10)	0.0319 (12)	0.0013 (9)	−0.0079 (10)	−0.0056 (9)
C16B	0.0457 (13)	0.0185 (10)	0.0256 (11)	0.0027 (8)	0.0056 (9)	−0.0001 (8)
C17B	0.0299 (10)	0.0180 (9)	0.0289 (11)	0.0043 (8)	0.0065 (9)	0.0025 (8)

### 1-Benzoyl-4-(4-nitrophenyl)piperazine (I) Geometric parameters (Å, º)

**Table:**

O1A—C11A	1.228 (2)	O1B—C11B	1.228 (2)
O2A—N3A	1.228 (2)	O2B—N3B	1.231 (2)
O3A—N3A	1.234 (2)	O3B—N3B	1.235 (2)
N1A—C11A	1.358 (2)	N1B—C11B	1.354 (3)
N1A—C4A	1.459 (2)	N1B—C4B	1.457 (3)
N1A—C1A	1.464 (2)	N1B—C1B	1.462 (2)
N2A—C5A	1.391 (2)	N2B—C5B	1.383 (3)
N2A—C3A	1.460 (2)	N2B—C2B	1.464 (2)
N2A—C2A	1.466 (2)	N2B—C3B	1.465 (2)
N3A—C8A	1.449 (3)	N3B—C8B	1.442 (3)
C1A—C2A	1.523 (3)	C1B—C2B	1.513 (3)
C1A—H1AA	0.9900	C1B—H1BA	0.9900
C1A—H1AB	0.9900	C1B—H1BB	0.9900
C2A—H2AA	0.9900	C2B—H2BA	0.9900
C2A—H2AB	0.9900	C2B—H2BB	0.9900
C3A—C4A	1.516 (3)	C3B—C4B	1.514 (3)
C3A—H3AA	0.9900	C3B—H3BA	0.9900
C3A—H3AB	0.9900	C3B—H3BB	0.9900
C4A—H4AA	0.9900	C4B—H4BA	0.9900
C4A—H4AB	0.9900	C4B—H4BB	0.9900
C5A—C6A	1.407 (3)	C5B—C10B	1.409 (3)
C5A—C10A	1.412 (3)	C5B—C6B	1.414 (3)
C6A—C7A	1.381 (3)	C6B—C7B	1.373 (3)
C6A—H6A	0.9500	C6B—H6B	0.9500
C7A—C8A	1.386 (3)	C7B—C8B	1.389 (3)
C7A—H7A	0.9500	C7B—H7B	0.9500
C8A—C9A	1.392 (3)	C8B—C9B	1.388 (3)
C9A—C10A	1.377 (3)	C9B—C10B	1.376 (3)
C9A—H9A	0.9500	C9B—H9B	0.9500
C10A—H10A	0.9500	C10B—H10B	0.9500
C11A—C12A	1.502 (3)	C11B—C12B	1.500 (3)
C12A—C13A	1.392 (3)	C12B—C17B	1.396 (3)
C12A—C17A	1.397 (3)	C12B—C13B	1.396 (3)
C13A—C14A	1.393 (3)	C13B—C14B	1.388 (3)
C13A—H13A	0.9500	C13B—H13B	0.9500
C14A—C15A	1.391 (3)	C14B—C15B	1.377 (3)
C14A—H14A	0.9500	C14B—H14B	0.9500
C15A—C16A	1.385 (3)	C15B—C16B	1.392 (4)
C15A—H15A	0.9500	C15B—H15B	0.9500
C16A—C17A	1.389 (3)	C16B—C17B	1.385 (3)
C16A—H16A	0.9500	C16B—H16B	0.9500
C17A—H17A	0.9500	C17B—H17B	0.9500
			
C11A—N1A—C4A	119.69 (15)	C11B—N1B—C4B	119.94 (16)
C11A—N1A—C1A	126.84 (16)	C11B—N1B—C1B	127.86 (17)
C4A—N1A—C1A	113.47 (15)	C4B—N1B—C1B	111.31 (16)
C5A—N2A—C3A	119.91 (16)	C5B—N2B—C2B	120.68 (15)
C5A—N2A—C2A	119.61 (16)	C5B—N2B—C3B	120.49 (16)
C3A—N2A—C2A	110.80 (15)	C2B—N2B—C3B	112.64 (16)
O2A—N3A—O3A	122.22 (18)	O2B—N3B—O3B	122.08 (17)
O2A—N3A—C8A	119.29 (16)	O2B—N3B—C8B	118.93 (15)
O3A—N3A—C8A	118.49 (17)	O3B—N3B—C8B	118.99 (17)
N1A—C1A—C2A	110.46 (16)	N1B—C1B—C2B	110.61 (16)
N1A—C1A—H1AA	109.6	N1B—C1B—H1BA	109.5
C2A—C1A—H1AA	109.6	C2B—C1B—H1BA	109.5
N1A—C1A—H1AB	109.6	N1B—C1B—H1BB	109.5
C2A—C1A—H1AB	109.6	C2B—C1B—H1BB	109.5
H1AA—C1A—H1AB	108.1	H1BA—C1B—H1BB	108.1
N2A—C2A—C1A	111.44 (16)	N2B—C2B—C1B	110.58 (16)
N2A—C2A—H2AA	109.3	N2B—C2B—H2BA	109.5
C1A—C2A—H2AA	109.3	C1B—C2B—H2BA	109.5
N2A—C2A—H2AB	109.3	N2B—C2B—H2BB	109.5
C1A—C2A—H2AB	109.3	C1B—C2B—H2BB	109.5
H2AA—C2A—H2AB	108.0	H2BA—C2B—H2BB	108.1
N2A—C3A—C4A	109.37 (16)	N2B—C3B—C4B	111.59 (17)
N2A—C3A—H3AA	109.8	N2B—C3B—H3BA	109.3
C4A—C3A—H3AA	109.8	C4B—C3B—H3BA	109.3
N2A—C3A—H3AB	109.8	N2B—C3B—H3BB	109.3
C4A—C3A—H3AB	109.8	C4B—C3B—H3BB	109.3
H3AA—C3A—H3AB	108.2	H3BA—C3B—H3BB	108.0
N1A—C4A—C3A	111.81 (15)	N1B—C4B—C3B	110.14 (16)
N1A—C4A—H4AA	109.3	N1B—C4B—H4BA	109.6
C3A—C4A—H4AA	109.3	C3B—C4B—H4BA	109.6
N1A—C4A—H4AB	109.3	N1B—C4B—H4BB	109.6
C3A—C4A—H4AB	109.3	C3B—C4B—H4BB	109.6
H4AA—C4A—H4AB	107.9	H4BA—C4B—H4BB	108.1
N2A—C5A—C6A	121.83 (17)	N2B—C5B—C10B	121.57 (18)
N2A—C5A—C10A	119.99 (17)	N2B—C5B—C6B	121.25 (16)
C6A—C5A—C10A	118.13 (17)	C10B—C5B—C6B	117.15 (18)
C7A—C6A—C5A	120.95 (17)	C7B—C6B—C5B	121.61 (17)
C7A—C6A—H6A	119.5	C7B—C6B—H6B	119.2
C5A—C6A—H6A	119.5	C5B—C6B—H6B	119.2
C6A—C7A—C8A	119.36 (19)	C6B—C7B—C8B	119.45 (18)
C6A—C7A—H7A	120.3	C6B—C7B—H7B	120.3
C8A—C7A—H7A	120.3	C8B—C7B—H7B	120.3
C7A—C8A—C9A	121.29 (19)	C9B—C8B—C7B	120.44 (19)
C7A—C8A—N3A	119.65 (18)	C9B—C8B—N3B	119.34 (17)
C9A—C8A—N3A	119.06 (17)	C7B—C8B—N3B	120.22 (17)
C10A—C9A—C8A	119.21 (17)	C10B—C9B—C8B	119.97 (18)
C10A—C9A—H9A	120.4	C10B—C9B—H9B	120.0
C8A—C9A—H9A	120.4	C8B—C9B—H9B	120.0
C9A—C10A—C5A	121.06 (18)	C9B—C10B—C5B	121.08 (19)
C9A—C10A—H10A	119.5	C9B—C10B—H10B	119.5
C5A—C10A—H10A	119.5	C5B—C10B—H10B	119.5
O1A—C11A—N1A	121.61 (18)	O1B—C11B—N1B	121.63 (19)
O1A—C11A—C12A	119.32 (17)	O1B—C11B—C12B	118.80 (18)
N1A—C11A—C12A	119.05 (16)	N1B—C11B—C12B	119.51 (17)
C13A—C12A—C17A	119.19 (18)	C17B—C12B—C13B	118.9 (2)
C13A—C12A—C11A	122.26 (17)	C17B—C12B—C11B	117.66 (17)
C17A—C12A—C11A	118.19 (16)	C13B—C12B—C11B	123.07 (18)
C12A—C13A—C14A	120.40 (18)	C14B—C13B—C12B	120.0 (2)
C12A—C13A—H13A	119.8	C14B—C13B—H13B	120.0
C14A—C13A—H13A	119.8	C12B—C13B—H13B	120.0
C15A—C14A—C13A	119.89 (19)	C15B—C14B—C13B	120.76 (19)
C15A—C14A—H14A	120.1	C15B—C14B—H14B	119.6
C13A—C14A—H14A	120.1	C13B—C14B—H14B	119.6
C16A—C15A—C14A	120.00 (18)	C14B—C15B—C16B	119.8 (2)
C16A—C15A—H15A	120.0	C14B—C15B—H15B	120.1
C14A—C15A—H15A	120.0	C16B—C15B—H15B	120.1
C15A—C16A—C17A	120.16 (18)	C17B—C16B—C15B	119.8 (2)
C15A—C16A—H16A	119.9	C17B—C16B—H16B	120.1
C17A—C16A—H16A	119.9	C15B—C16B—H16B	120.1
C16A—C17A—C12A	120.32 (18)	C16B—C17B—C12B	120.77 (19)
C16A—C17A—H17A	119.8	C16B—C17B—H17B	119.6
C12A—C17A—H17A	119.8	C12B—C17B—H17B	119.6
			
C11A—N1A—C1A—C2A	−129.76 (19)	C11B—N1B—C1B—C2B	−132.2 (2)
C4A—N1A—C1A—C2A	51.1 (2)	C4B—N1B—C1B—C2B	58.7 (2)
C5A—N2A—C2A—C1A	−155.27 (17)	C5B—N2B—C2B—C1B	−154.05 (17)
C3A—N2A—C2A—C1A	58.7 (2)	C3B—N2B—C2B—C1B	53.5 (2)
N1A—C1A—C2A—N2A	−53.4 (2)	N1B—C1B—C2B—N2B	−55.6 (2)
C5A—N2A—C3A—C4A	155.04 (17)	C5B—N2B—C3B—C4B	154.12 (18)
C2A—N2A—C3A—C4A	−59.0 (2)	C2B—N2B—C3B—C4B	−53.3 (2)
C11A—N1A—C4A—C3A	127.48 (18)	C11B—N1B—C4B—C3B	132.20 (19)
C1A—N1A—C4A—C3A	−53.3 (2)	C1B—N1B—C4B—C3B	−57.7 (2)
N2A—C3A—C4A—N1A	56.2 (2)	N2B—C3B—C4B—N1B	54.6 (2)
C3A—N2A—C5A—C6A	4.6 (3)	C2B—N2B—C5B—C10B	9.5 (3)
C2A—N2A—C5A—C6A	−138.34 (19)	C3B—N2B—C5B—C10B	159.88 (19)
C3A—N2A—C5A—C10A	−172.59 (16)	C2B—N2B—C5B—C6B	−172.66 (17)
C2A—N2A—C5A—C10A	44.4 (3)	C3B—N2B—C5B—C6B	−22.3 (3)
N2A—C5A—C6A—C7A	−176.91 (18)	N2B—C5B—C6B—C7B	−172.84 (18)
C10A—C5A—C6A—C7A	0.4 (3)	C10B—C5B—C6B—C7B	5.0 (3)
C5A—C6A—C7A—C8A	0.2 (3)	C5B—C6B—C7B—C8B	−0.9 (3)
C6A—C7A—C8A—C9A	−0.3 (3)	C6B—C7B—C8B—C9B	−3.6 (3)
C6A—C7A—C8A—N3A	−179.79 (17)	C6B—C7B—C8B—N3B	176.42 (17)
O2A—N3A—C8A—C7A	−4.2 (3)	O2B—N3B—C8B—C9B	−173.79 (18)
O3A—N3A—C8A—C7A	174.95 (19)	O3B—N3B—C8B—C9B	5.5 (3)
O2A—N3A—C8A—C9A	176.25 (18)	O2B—N3B—C8B—C7B	6.2 (3)
O3A—N3A—C8A—C9A	−4.6 (3)	O3B—N3B—C8B—C7B	−174.52 (18)
C7A—C8A—C9A—C10A	−0.1 (3)	C7B—C8B—C9B—C10B	3.9 (3)
N3A—C8A—C9A—C10A	179.36 (17)	N3B—C8B—C9B—C10B	−176.2 (2)
C8A—C9A—C10A—C5A	0.7 (3)	C8B—C9B—C10B—C5B	0.4 (3)
N2A—C5A—C10A—C9A	176.52 (17)	N2B—C5B—C10B—C9B	173.1 (2)
C6A—C5A—C10A—C9A	−0.8 (3)	C6B—C5B—C10B—C9B	−4.8 (3)
C4A—N1A—C11A—O1A	−12.0 (3)	C4B—N1B—C11B—O1B	0.7 (3)
C1A—N1A—C11A—O1A	168.91 (18)	C1B—N1B—C11B—O1B	−167.5 (2)
C4A—N1A—C11A—C12A	166.20 (16)	C4B—N1B—C11B—C12B	−176.48 (16)
C1A—N1A—C11A—C12A	−12.9 (3)	C1B—N1B—C11B—C12B	15.3 (3)
O1A—C11A—C12A—C13A	131.5 (2)	O1B—C11B—C12B—C17B	40.6 (3)
N1A—C11A—C12A—C13A	−46.8 (3)	N1B—C11B—C12B—C17B	−142.11 (18)
O1A—C11A—C12A—C17A	−41.6 (2)	O1B—C11B—C12B—C13B	−131.9 (2)
N1A—C11A—C12A—C17A	140.11 (18)	N1B—C11B—C12B—C13B	45.4 (3)
C17A—C12A—C13A—C14A	−1.3 (3)	C17B—C12B—C13B—C14B	0.6 (3)
C11A—C12A—C13A—C14A	−174.32 (17)	C11B—C12B—C13B—C14B	173.05 (18)
C12A—C13A—C14A—C15A	0.0 (3)	C12B—C13B—C14B—C15B	−0.7 (3)
C13A—C14A—C15A—C16A	0.7 (3)	C13B—C14B—C15B—C16B	0.3 (3)
C14A—C15A—C16A—C17A	−0.1 (3)	C14B—C15B—C16B—C17B	0.2 (3)
C15A—C16A—C17A—C12A	−1.2 (3)	C15B—C16B—C17B—C12B	−0.3 (3)
C13A—C12A—C17A—C16A	1.9 (3)	C13B—C12B—C17B—C16B	−0.2 (3)
C11A—C12A—C17A—C16A	175.23 (16)	C11B—C12B—C17B—C16B	−172.98 (18)

### 1-Benzoyl-4-(4-nitrophenyl)piperazine (I) Hydrogen-bond geometry (Å, º)

**Table:**

*D*—H···*A*	*D*—H	H···*A*	*D*···*A*	*D*—H···*A*
C6*B*—H6*B*···O1*A*	0.95	2.50	3.140 (2)	125
C7*B*—H7*B*···O1*A*	0.95	2.58	3.171 (2)	120
C6*A*—H6*A*···O1*B*^i^	0.95	2.47	3.173 (2)	131
C7*A*—H7*A*···O1*B*^i^	0.95	2.78	3.317 (2)	117

Symmetry code: (i) *x*, *y*, *z*+1.

### 1-(4-Bromobenzoyl)-4-phenylpiperazine (II) Crystal data

**Table:**

C_17_H_17_BrN_2_O	*F*(000) = 352
*M**_r_* = 345.23	*D*_x_ = 1.605 Mg m^−^^3^
Monoclinic, *P*2_1_	Mo *K*α radiation, λ = 0.71073 Å
*a* = 7.5162 (3) Å	Cell parameters from 9856 reflections
*b* = 6.1125 (2) Å	θ = 2.6–27.5°
*c* = 15.7249 (5) Å	µ = 2.88 mm^−^^1^
β = 98.625 (1)°	*T* = 90 K
*V* = 714.28 (4) Å^3^	Slab cut from lath, colourless
*Z* = 2	0.35 × 0.20 × 0.06 mm

### 1-(4-Bromobenzoyl)-4-phenylpiperazine (II) Data collection

**Table:**

Bruker D8 Venture dual source diffractometer	6918 independent reflections
Radiation source: microsource	6410 reflections with *I* > 2σ(*I*)
Detector resolution: 7.41 pixels mm^-1^	*R*_int_ = 0.065
φ and ω scans	θ_max_ = 27.5°, θ_min_ = 2.6°
Absorption correction: multi-scan (*TWINABS*; Sheldrick, 2012)	*h* = −9→9
*T*_min_ = 0.568, *T*_max_ = 0.806	*k* = −7→7
6918 measured reflections	*l* = −20→20

### 1-(4-Bromobenzoyl)-4-phenylpiperazine (II) Refinement

**Table:**

Refinement on *F*^2^	Secondary atom site location: difference Fourier map
Least-squares matrix: full	Hydrogen site location: difference Fourier map
*R*[*F*^2^ > 2σ(*F*^2^)] = 0.023	H-atom parameters constrained
*wR*(*F*^2^) = 0.049	*w* = 1/[σ^2^(*F*_o_^2^) + (0.0158*P*)^2^ + 0.0999*P*] where *P* = (*F*_o_^2^ + 2*F*_c_^2^)/3
*S* = 1.04	(Δ/σ)_max_ = 0.001
6918 reflections	Δρ_max_ = 0.29 e Å^−^^3^
191 parameters	Δρ_min_ = −0.22 e Å^−^^3^
1 restraint	Absolute structure: Flack *x* determined using 1306 quotients [(*I*^+^)-(*I*^-^)]/[(*I*^+^)+(*I*^-^)] (Parsons *et al.*, 2013)
Primary atom site location: structure-invariant direct methods	Absolute structure parameter: 0.012 (4)

### 1-(4-Bromobenzoyl)-4-phenylpiperazine (II) Special details

**Table:**

Geometry. All esds (except the esd in the dihedral angle between two l.s. planes) are estimated using the full covariance matrix. The cell esds are taken into account individually in the estimation of esds in distances, angles and torsion angles; correlations between esds in cell parameters are only used when they are defined by crystal symmetry. An approximate (isotropic) treatment of cell esds is used for estimating esds involving l.s. planes.
Refinement. Refinement progress was checked using *Platon* (Spek, 2020) and by an *R*-tensor (Parkin, 2000). The final model was further checked with the IUCr utility *checkCIF*. Refined as a 2-component aggregate.

### 1-(4-Bromobenzoyl)-4-phenylpiperazine (II) Fractional atomic coordinates and isotropic or equivalent isotropic displacement parameters (Å^2^)

**Table:**

	*x*	*y*	*z*	*U*_iso_*/*U*_eq_	
Br1	0.09055 (3)	0.11286 (6)	0.95039 (2)	0.01878 (8)	
O1	0.6730 (3)	0.8844 (4)	0.83992 (15)	0.0238 (5)	
N1	0.7763 (3)	0.6022 (7)	0.76896 (13)	0.0138 (4)	
N2	0.8022 (3)	0.5773 (4)	0.58908 (14)	0.0128 (6)	
C1	0.7544 (4)	0.3913 (5)	0.72408 (19)	0.0170 (6)	
H1A	0.666366	0.300535	0.749181	0.020*	
H1B	0.870892	0.312654	0.731847	0.020*	
C2	0.6896 (4)	0.4248 (5)	0.62881 (19)	0.0170 (6)	
H2A	0.688355	0.281887	0.599077	0.020*	
H2B	0.564666	0.480861	0.621072	0.020*	
C3	0.8331 (4)	0.7843 (5)	0.63607 (18)	0.0151 (6)	
H3A	0.719803	0.869390	0.629414	0.018*	
H3B	0.923829	0.871335	0.611219	0.018*	
C4	0.8985 (4)	0.7459 (5)	0.73118 (19)	0.0153 (6)	
H4A	1.019905	0.679267	0.738344	0.018*	
H4B	0.907757	0.887866	0.761819	0.018*	
C5	0.7688 (3)	0.5918 (7)	0.49830 (16)	0.0139 (6)	
C6	0.8200 (4)	0.7753 (5)	0.4548 (2)	0.0187 (6)	
H6	0.874536	0.895931	0.486795	0.022*	
C7	0.7922 (5)	0.7840 (5)	0.3655 (2)	0.0211 (7)	
H7	0.828030	0.910876	0.337573	0.025*	
C8	0.7134 (3)	0.6116 (10)	0.31631 (16)	0.0204 (5)	
H8	0.694672	0.618576	0.255269	0.024*	
C9	0.6633 (5)	0.4301 (6)	0.3587 (2)	0.0234 (7)	
H9	0.610006	0.309579	0.326239	0.028*	
C10	0.6889 (5)	0.4190 (5)	0.4478 (2)	0.0218 (7)	
H10	0.651625	0.291940	0.475097	0.026*	
C11	0.6668 (4)	0.6894 (5)	0.82122 (18)	0.0146 (6)	
C12	0.5338 (4)	0.5444 (4)	0.85605 (17)	0.0135 (6)	
C13	0.5770 (4)	0.3395 (5)	0.89205 (18)	0.0128 (6)	
H13	0.696220	0.285267	0.894978	0.015*	
C14	0.4476 (4)	0.2132 (5)	0.92378 (17)	0.0134 (6)	
H14	0.477362	0.073859	0.948719	0.016*	
C15	0.2748 (4)	0.2952 (5)	0.91818 (18)	0.0138 (6)	
C16	0.2290 (4)	0.5014 (5)	0.88668 (18)	0.0148 (6)	
H16	0.110701	0.556801	0.885896	0.018*	
C17	0.3610 (3)	0.6267 (8)	0.85598 (15)	0.0153 (5)	
H17	0.332668	0.769940	0.834754	0.018*	

### 1-(4-Bromobenzoyl)-4-phenylpiperazine (II) Atomic displacement parameters (Å^2^)

**Table:**

	*U*^11^	*U*^22^	*U*^33^	*U*^12^	*U*^13^	*U*^23^
Br1	0.01541 (13)	0.01942 (13)	0.02254 (13)	−0.0012 (2)	0.00626 (9)	0.00283 (19)
O1	0.0269 (12)	0.0140 (11)	0.0336 (13)	−0.0023 (9)	0.0145 (11)	−0.0065 (9)
N1	0.0159 (10)	0.0124 (10)	0.0136 (9)	−0.0026 (16)	0.0042 (8)	−0.0007 (16)
N2	0.0162 (11)	0.0102 (17)	0.0123 (10)	−0.0017 (9)	0.0032 (8)	−0.0013 (10)
C1	0.0258 (17)	0.0119 (14)	0.0149 (15)	−0.0002 (12)	0.0081 (13)	−0.0008 (12)
C2	0.0255 (17)	0.0119 (14)	0.0140 (15)	−0.0060 (12)	0.0050 (12)	−0.0023 (11)
C3	0.0161 (16)	0.0129 (14)	0.0159 (15)	−0.0020 (11)	0.0016 (12)	0.0001 (12)
C4	0.0141 (15)	0.0179 (15)	0.0146 (14)	−0.0037 (11)	0.0044 (12)	0.0001 (11)
C5	0.0101 (11)	0.0178 (17)	0.0136 (11)	0.0018 (15)	0.0010 (9)	0.0042 (15)
C6	0.0194 (17)	0.0190 (16)	0.0179 (16)	−0.0020 (13)	0.0030 (13)	0.0020 (12)
C7	0.0218 (18)	0.0223 (17)	0.0202 (16)	−0.0008 (13)	0.0066 (13)	0.0074 (13)
C8	0.0167 (12)	0.0307 (14)	0.0134 (11)	0.004 (2)	0.0013 (9)	0.002 (2)
C9	0.0257 (19)	0.0272 (18)	0.0167 (16)	−0.0065 (14)	0.0010 (13)	−0.0038 (13)
C10	0.0264 (19)	0.0216 (17)	0.0166 (16)	−0.0074 (13)	0.0009 (13)	0.0012 (13)
C11	0.0146 (14)	0.0155 (13)	0.0130 (13)	0.002 (1)	0.0001 (11)	0.0007 (10)
C12	0.0154 (14)	0.0151 (15)	0.0097 (13)	−0.0001 (10)	0.0007 (11)	−0.0019 (9)
C13	0.0127 (14)	0.0143 (14)	0.0115 (13)	0.0026 (11)	0.0017 (11)	−0.0019 (11)
C14	0.0173 (15)	0.0132 (14)	0.0095 (13)	0.0011 (11)	0.0010 (11)	−0.0014 (11)
C15	0.0135 (14)	0.0174 (15)	0.0110 (14)	−0.0032 (11)	0.0035 (11)	−0.0015 (11)
C16	0.0122 (14)	0.0186 (15)	0.0138 (14)	0.0029 (11)	0.0029 (11)	0.0007 (12)
C17	0.0194 (12)	0.0145 (14)	0.0117 (11)	0.0030 (19)	0.0012 (9)	0.0014 (16)

### 1-(4-Bromobenzoyl)-4-phenylpiperazine (II) Geometric parameters (Å, º)

**Table:**

Br1—C15	1.904 (3)	C6—C7	1.389 (4)
O1—C11	1.227 (4)	C6—H6	0.9500
N1—C11	1.356 (4)	C7—C8	1.387 (6)
N1—C4	1.460 (4)	C7—H7	0.9500
N1—C1	1.467 (4)	C8—C9	1.376 (6)
N2—C5	1.415 (3)	C8—H8	0.9500
N2—C2	1.461 (4)	C9—C10	1.387 (4)
N2—C3	1.466 (4)	C9—H9	0.9500
C1—C2	1.518 (4)	C10—H10	0.9500
C1—H1A	0.9900	C11—C12	1.500 (4)
C1—H1B	0.9900	C12—C17	1.393 (4)
C2—H2A	0.9900	C12—C13	1.393 (4)
C2—H2B	0.9900	C13—C14	1.391 (4)
C3—C4	1.521 (4)	C13—H13	0.9500
C3—H3A	0.9900	C14—C15	1.383 (4)
C3—H3B	0.9900	C14—H14	0.9500
C4—H4A	0.9900	C15—C16	1.379 (4)
C4—H4B	0.9900	C16—C17	1.395 (5)
C5—C6	1.397 (5)	C16—H16	0.9500
C5—C10	1.401 (5)	C17—H17	0.9500
			
C11—N1—C4	119.1 (3)	C5—C6—H6	119.5
C11—N1—C1	127.0 (3)	C8—C7—C6	121.5 (3)
C4—N1—C1	111.4 (2)	C8—C7—H7	119.3
C5—N2—C2	116.4 (2)	C6—C7—H7	119.3
C5—N2—C3	116.4 (3)	C9—C8—C7	117.9 (2)
C2—N2—C3	113.2 (2)	C9—C8—H8	121.1
N1—C1—C2	110.6 (2)	C7—C8—H8	121.1
N1—C1—H1A	109.5	C8—C9—C10	121.5 (3)
C2—C1—H1A	109.5	C8—C9—H9	119.3
N1—C1—H1B	109.5	C10—C9—H9	119.3
C2—C1—H1B	109.5	C9—C10—C5	121.2 (3)
H1A—C1—H1B	108.1	C9—C10—H10	119.4
N2—C2—C1	112.8 (2)	C5—C10—H10	119.4
N2—C2—H2A	109.0	O1—C11—N1	121.5 (3)
C1—C2—H2A	109.0	O1—C11—C12	119.4 (3)
N2—C2—H2B	109.0	N1—C11—C12	119.1 (3)
C1—C2—H2B	109.0	C17—C12—C13	119.1 (3)
H2A—C2—H2B	107.8	C17—C12—C11	117.3 (3)
N2—C3—C4	111.4 (2)	C13—C12—C11	123.6 (3)
N2—C3—H3A	109.3	C14—C13—C12	120.8 (3)
C4—C3—H3A	109.3	C14—C13—H13	119.6
N2—C3—H3B	109.3	C12—C13—H13	119.6
C4—C3—H3B	109.3	C15—C14—C13	118.5 (3)
H3A—C3—H3B	108.0	C15—C14—H14	120.8
N1—C4—C3	111.3 (2)	C13—C14—H14	120.8
N1—C4—H4A	109.4	C16—C15—C14	122.4 (3)
C3—C4—H4A	109.4	C16—C15—Br1	118.6 (2)
N1—C4—H4B	109.4	C14—C15—Br1	119.0 (2)
C3—C4—H4B	109.4	C15—C16—C17	118.3 (3)
H4A—C4—H4B	108.0	C15—C16—H16	120.9
C6—C5—C10	117.0 (2)	C17—C16—H16	120.9
C6—C5—N2	121.6 (3)	C12—C17—C16	120.9 (4)
C10—C5—N2	121.4 (3)	C12—C17—H17	119.6
C7—C6—C5	121.0 (3)	C16—C17—H17	119.6
C7—C6—H6	119.5		
			
C11—N1—C1—C2	105.4 (3)	C6—C5—C10—C9	0.4 (5)
C4—N1—C1—C2	−56.4 (3)	N2—C5—C10—C9	−177.2 (3)
C5—N2—C2—C1	170.4 (3)	C4—N1—C11—O1	−2.8 (4)
C3—N2—C2—C1	−50.7 (3)	C1—N1—C11—O1	−163.3 (3)
N1—C1—C2—N2	52.8 (3)	C4—N1—C11—C12	176.5 (2)
C5—N2—C3—C4	−170.3 (2)	C1—N1—C11—C12	16.0 (4)
C2—N2—C3—C4	50.8 (3)	O1—C11—C12—C17	42.2 (4)
C11—N1—C4—C3	−105.9 (3)	N1—C11—C12—C17	−137.1 (3)
C1—N1—C4—C3	57.5 (3)	O1—C11—C12—C13	−134.3 (3)
N2—C3—C4—N1	−54.1 (3)	N1—C11—C12—C13	46.4 (4)
C2—N2—C5—C6	158.2 (3)	C17—C12—C13—C14	3.2 (4)
C3—N2—C5—C6	20.7 (4)	C11—C12—C13—C14	179.6 (3)
C2—N2—C5—C10	−24.3 (4)	C12—C13—C14—C15	0.5 (4)
C3—N2—C5—C10	−161.8 (3)	C13—C14—C15—C16	−3.6 (4)
C10—C5—C6—C7	0.0 (5)	C13—C14—C15—Br1	173.6 (2)
N2—C5—C6—C7	177.6 (3)	C14—C15—C16—C17	2.9 (4)
C5—C6—C7—C8	−0.2 (5)	Br1—C15—C16—C17	−174.3 (2)
C6—C7—C8—C9	−0.1 (5)	C13—C12—C17—C16	−3.8 (4)
C7—C8—C9—C10	0.5 (5)	C11—C12—C17—C16	179.5 (3)
C8—C9—C10—C5	−0.7 (5)	C15—C16—C17—C12	0.9 (4)

### 1-(4-Bromobenzoyl)-4-phenylpiperazine (II) Hydrogen-bond geometry (Å, º)

**Table:**

*D*—H···*A*	*D*—H	H···*A*	*D*···*A*	*D*—H···*A*
C13—H13···O1^i^	0.95	2.60	3.018 (4)	107
C14—H14···O1^i^	0.95	2.68	3.052 (4)	104

Symmetry code: (i) *x*, *y*−1, *z*.
